# Exploring the Variability in Acute Glycemic Responses to Exercise in Type 2 Diabetes

**DOI:** 10.1155/2013/591574

**Published:** 2013-07-29

**Authors:** Tasuku Terada, Alanna Friesen, Baljot S. Chahal, Gordon J. Bell, Linda J. McCargar, Normand G. Boulé

**Affiliations:** ^1^Faculty of Physical Education & Recreation, University of AB, 1-059 Li Ka Shing Center, Edmonton, AB, Canada T6G 2H9; ^2^Department of Agricultural, Food and Nutritional Science, University of AB, 2-012D Li Ka Shing Center, Health Research Innovation, Edmonton, AB, Canada T6G 2H9

## Abstract

*Aim*. To explore the factors associated with exercise-induced acute capillary glucose (CapBG) changes in individuals with type 2 diabetes (T2D). *Methods*. Fifteen individuals with T2D were randomly assigned to energy-matched high intensity interval exercise (HI-IE) and moderate intensity continuous exercise (MI-CE) interventions and performed a designated exercise protocol 5 days per week for 12 weeks. The duration of exercise progressed from 30 to 60 minutes. CapBG was measured immediately before and after each exercise session. Timing of food and antihyperglycemic medication intake prior to exercise was recorded. *Results*. Overall, the mean CapBG was lowered by 1.9 mmol/L (*P* < 0.001) with the change ranging from −8.9 to +2.7 mmol/L. Preexercise CapBG (44%; *P* < 0.001), medication (5%; *P* < 0.001), food intake (4%; *P* = 0.043), exercise duration (5%; *P* < 0.001), and exercise intensity (1%; *P* = 0.007) were all associated with CapBG changes, explaining 59% of the variability. *Conclusion*. The greater reduction in CapBG seen in individuals with higher preexercise CapBG may suggest the importance of exercise in the population with elevated glycemia. Lower blood glucose can be achieved with moderate intensity exercise, but prolonging exercise duration and/or including brief bouts of intense exercise accentuate the reduction, which can further be magnified by performing exercise after meals and antihyperglycemic medication. This trial is registered with ClinicalTrial.gov NCT01144078.

## 1. Introduction

One of the major goals of prescribing exercise for individuals with type 2 diabetes (T2D) is to reduce hyperglycemia, a risk factor for long-term complications. There have been several meta-analyses demonstrating that, on average, exercise has a clinically meaningful impact on glycemic control in individuals with T2D [1–3]. However, while the overall glucose-lowering effect of exercise is well recognized, large glycemic heterogeneity among studies and within individuals is often under appreciated. Indeed, there are divergent findings as to which characteristics best predict long-term improvements in glycemic control. One systematic review showed that exercise volume was the major determinant of glycemic changes in response to exercise training [4], while another showed exercise intensity is more closely associated [5]. 

The long-term glycemic benefit of exercise training is considered as the sum of the effects of each successive bout of exercise [6]. Accordingly, an enhanced understanding of the heterogeneous acute responses to exercise may elucidate the varied degree of training effects. To date, a number of studies have examined the acute effects of different exercise interventions on blood glucose and, on the basis of these results, it is generally agreed that moderate intensity exercise reduces blood glucose [7, 8]. Glycemic responses to high intensity exercise, on the other hand, are inconsistent with some studies showing a greater reduction than moderate intensity exercise [8, 9] while another showing markedly increased glucose concentration [10]. Some researchers also argued that total exercise volume independent of exercise intensity determines the degree of glycemic reduction [11, 12]. Consequently, although exercise is an important factor for better glucoregulation, it is not clear how different exercise interventions acutely influence blood glucose. 

While the contribution of different exercise interventions to the heterogeneity of glycemic responses remains to be clarified, available evidence suggests that the timing of exercise in relation to meals and medications also need to be considered. For example, Poirier et al. showed that moderate intensity exercise performed after a meal elicits a meaningful decrease in blood glucose but results in little change if performed under the fasting condition [13]. Furthermore, the interactive effects of oral antihyperglycemic medication and exercise on blood glucose reduction have been suggested [14]. Collectively, it can be speculated that glycemic responses to exercise are the result of a complex interplay between external factors that influence blood glucose concentrations and the effects of different exercise interventions. 

Given that exercise is often performed at different times of the day when the influence of medication and food intake can vary, it is of importance to investigate the effect of the exercise intervention in conjunction with the timing of exercise. Elucidating the factors associated with varied glycemic responses may help explain heterogeneity in effect sizes and, thereby, lead to the development of more effective implementation of exercise interventions. The purpose of this study was to simultaneously investigate the effects of different exercise interventions and external factors known to influence exercise-induced changes in glucose concentration. 

## 2. Methods

### 2.1. Participants

Post hoc analysis of a study examining the effects of high intensity interval exercise (HI-IE) and moderate intensity continuous exercise (MI-CE) on glycosylated hemoglobin (A1c) and abdominal fat [15] was conducted. In brief, participants were required to be diagnosed with T2D, 55–75 years of age, nonsmokers, relatively sedentary (<150 minutes of structured exercise per week), and able to exercise 5 days per week (see [15] for complete details). Participants meeting the criteria had their blood pressure (BP) measured, and those with BP < 140/90 mmHg performed a graded exercise stress test under the supervision of a physician to confirm the absence of any underlying contraindications to performing high intensity exercise. All participants provided written informed consent. Ethical approval was obtained from the University of Alberta Health Research Ethics Board.

### 2.2. Baseline Measurement

Detailed baseline assessment was described elsewhere [15]. Briefly, eligible participants reported to laboratories at the University of Alberta to assess baseline anthropometric characteristics and peak oxygen consumption (VO_2peak_). VO_2peak_ was determined using a cycle ergometer (Monark 818; Monark, Varberg, Sweden) and a TrueMax (ParvoMedics, Sandy, UT) metabolic measurement system that was calibrated for air volume and gas concentration according to the manufacturer's instruction. 

### 2.3. Study Protocol

The exercise intervention was comprised of a 2-week run-in period followed by a 12-week training period. The run-in period was to habituate the participants to the exercise intervention protocols and to assess their compliance. A total of 6 exercise sessions (3 sessions per week) were held during the run-in period, and participants were instructed to complete a minimum of 5 sessions to be eligible for the study. The sessions alternated daily between stationary cycling and treadmill walking for 30 minutes at an exercise intensity corresponding to 40% of individually determined oxygen consumption reserve (VO_2_R) [16]. Appropriate intensity was prescribed by adjusting the speed and slope on the treadmill, or power output on the stationary cycle. 

 After the 2-week run-in period, the participants were randomly assigned (stratified by sex) to the HI-IE and MI-CE training interventions, which were matched for exercise duration, frequency, and average relative intensity. Stationary cycling and treadmill walking were alternated daily for exercise variety. Participants were required to complete an entire exercise session on either bike or treadmill and could not alternate on a given day. The MI-CE group performed continuous exercise at 40% VO_2_R, whereas the HI-IE group repetitively performed a 1 minute interval at 100% VO_2_R followed by 3 minutes at 20% VO_2_R on Monday through Friday with the exception of Wednesday, when they performed MI-CE protocol. The duration of exercise was 30, 45, and 60 minutes per session for weeks 1–4, weeks 5–8, and weeks 9–12, respectively. Both groups exercised at the time of participants' convenience 5 days per week for 12 consecutive weeks. All training sessions were supervised. 

### 2.4. Daily Measurement

Upon arrival to the fitness center for their exercise session, participants reported the timing of their most recent food intake and oral anti-hyperglycemic medication intake. The timing of events was chosen by the participants and not influenced by the investigators. A single CapBG was measured immediately (<10 minutes) before and after each exercise bout with a validated [17] One Touch Ultra 2 (LifeScan Milpitas, CA USA) handheld glucose monitor. Briefly, a finger was cleaned with an alcohol pad, allowed to dry, and then was pricked with a disposable lancet. The first drop of blood was wiped off with gauze, and the second drop was applied to the strip. 

### 2.5. Data Analyses

Due to interdependent nature of the data, raw CapBG data from each participant were stratified according to exercise modality (bike versus treadmill), exercise intensity, exercise duration, medication, and food intake. Mean blood glucose concentrations from each stratum were used for analysis.

 The interval between the individual exercise bout and the most recent meal intake was stratified into <2 hours, 2–6 hours, and >6 hours prior to exercise, while the time interval from the most recent oral anti-hyperglycemic medication was stratified into ≤6 hours and >6 hours. The cutoffs for food intake and medication were determined, respectively, from previous observations which revealed that hyperglycemia was most prominent for the first 2 hours subsequent to meal intake and remained elevated for the next 4 hours [18, 19] and from the plasma elimination half-life of metformin [20], the most commonly used medication in these participants.

 Treatment group differences in baseline characteristics were tested using independent *t*-test. Dependent *t*-test was used to compare pre- and postexercise CapBG concentrations. To determine the independent association of exercise modality, exercise intensity, exercise duration, food intake, medication, and preexercise CapBG concentrations on exercise-induced CapBG changes, multiple regression analysis was performed. Categorical data were dummy coded for the analysis. Analysis of covariance (ANCOVA) with preexercise CapBG used as a covariate was also performed to assess if there were any interaction effects between external factors and exercise interventions. 

 Data are presented as mean ± standard deviation unless otherwise stated. All statistical tests were two-tailed and *P* values of <0.05 were considered significant. Normality of the data and lack of multicollinearity were examined by investigating the distributions of residuals and by variance inflation factor, respectively. Statistical analyses were performed with Minitab 15 statistical software (Minitab Inc., State College, PA, USA). 

## 3. Results

### 3.1. Participants

Seven participants in HI-IE group and 8 in MI-CE completed all phases of the study. No severe adverse effect of exercise was observed, and no participants dropped out from the training program once they were randomized. Adherence rates for the group mean attendance were 61 sessions for HI-IE and 62 for MI-CE, respectively, (>97% of eligible exercise sessions for both groups). Descriptive characteristics of the 15 participants (8 males and 7 females) are summarized in Table 1. 

### 3.2. Glycemic Responses to Acute Exercise

**Figure 1:**
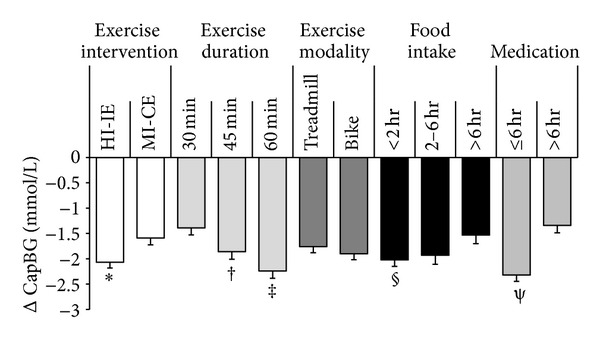
Effects of exercise intervention, exercise duration, exercise modality, food intake, and medication on exercise-induced CapBG reduction. Values are least square mean ± SE. *HI-IE (*P* = 0.007), ^†^45 min exercise (*P* = 0.015), ^‡^60 min exercise (*P* < 0.001), ^§^food intake <2 hours (*P* = 0.043), and ^*ψ*^medication ≤6 hours of exercise (*P* < 0.001) were all associated with greater CapBG reduction. HI-IE: high intensity interval exercise; MI-CE: moderate intensity continuous exercise; CapBG: capillary blood glucose.

**Figure 2:**
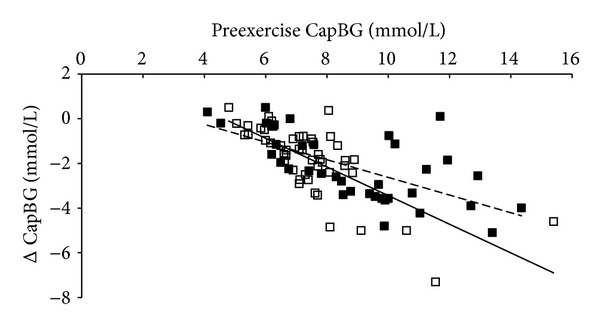
Correlations between preexercise CapBG and exercise-induced CapBG changes for both HI-IE and MI-CE. Filled and open squares represent MI-CE and HI-IE, respectively. Dotted and straight lines are regression lines of MI-CE and HI-IE, respectively. Glucose data obtained from each participant are categorized based on exercise intensity, duration, and modality, as well as food and medication status. Each box in the figure represents the mean glucose value obtained from each participant under different conditions. HI-IE: high intensity interval exercise; MI-CE: moderate intensity continuous exercise; CapBG: capillary blood glucose.

In total, 730 pre- and 730 postexercise CapBG measures were obtained. Preexercise CapBG did not change over the course of either training intervention and was consistently higher in MI-CE (*P* < 0.001). Overall changes in CapBG induced by exercise were significant (−1.9 ± 1.7 mmol; *P* < 0.001). However, despite the overall glucose-lowering effect of exercise, the degree of changes was highly heterogeneous, ranging from an 8.9 mmol/L reduction to a 2.7 mmol/L increase. Multiple regression analysis revealed that higher preexercise CapBG (44%; *P* < 0.001), anti-hyperglycemic medication within 6 hours of exercise (5%; *P* < 0.001), food intake within 2 hours of exercise (4%, *P* = 0.043), longer exercise duration (5%; *P* = 0.010), and high exercise intensity (1%; *P* = 0.007) were all associated with greater CapBG reduction, explaining 59% of the total variability (*P* < 0.001; Figure 1). Mean preexercise CapBG and changes in CapBG obtained for each participant under different conditions were plotted to schematically present the effects of preexercise CapBG on glucose concentration changes (Figure 2). Variance inflation factors among the independent variables were low (<2.3), indicating small degree of multicollinearity among the variables. Repeated ANCOVA consistently indicated significant effects of above variables on CapBG changes induced by exercise; however, no significant interaction effects existed between exercise and food or medication. 

## 4. Discussion

 This study examined the factors associated with heterogeneous CapBG responses to exercise and their individual contribution to the exercise-induced CapBG reduction in individuals with T2D. The primary finding of this study is that variability in the acute glycemic response to exercise can in large part be explained by easily acquired variables such as preexercise glucose concentrations. In addition, while higher preexercise CapBG was the strongest determinant of exercise-induced CapBG changes, our results also showed that longer exercise duration and higher exercise intensity, as well as anti-hyperglycemic medication and food intake prior to exercise, magnify the reduction in CapBG. 

 The correlation between preexercise CapBG and CapBG change observed in the present study is in line with the finding of Jeng et al. in which higher preexercise CapBG was most strongly associated with a greater reduction in CapBG after exercise followed by exercise duration and intensity, explaining 37% of the variance [8]. By including the external factors such as timing of medication and food intake, however, we showed that our model explains more variance (59%) associated with exercise. Furthermore, because the previous study [8] did not take exercise volume into account, it was not clear whether the effect was due to exercise intensity *per se* or due to greater total exercise volume that accompanies higher exercise intensity. The setting in our study where the exercise volume was equated between 2 exercise intervention groups allowed us to investigate the effect of exercise intensity independent of exercise volume.

 Our results demonstrated that, although its contribution to overall change in CapBG is small, higher exercise intensity results in greater reduction in CapBG than moderate intensity exercise matched for exercise volume. The MI-CE group had higher BMI at baseline, which may explain higher fasting blood glucose and preexercise CapBG. After adjusting for the preexercise CapBG, however, our study demonstrated that high intensity exercise lowers CapBG significantly more than MI-CE. This finding contradicts with previous studies showing that the effect of exercise on blood glucose reduction is related to exercise volume but not to exercise intensity [11, 12]. Because, unlike these previous studies where energy demand was matched by altering exercise duration, we matched exercise volume and also exercise duration between the two interventions, different exercise interventions may explain the divergent glycemic responses reported. Our finding builds on previous studies indicating potential superior benefits of HI-IE on body composition [21] and insulin sensitivity [22] to isocaloric moderate intensity exercise and suggests that HI-IE may also confer an additional benefit in terms of acute glycemic regulation.

 The association between longer exercise duration and a greater CapBG decline observed in this study is in accordance with previous studies [7, 8]. Enhanced direct oxidation of excessive blood glucose associated with greater exercise volume is likely to be responsible for greater reduction in CapBG. Likewise, small difference in calculated exercise volume (0.7 KJ·min^−1^ difference) may explain the lack of difference in CapBG responses between bike and treadmill. Collectively, our results suggest that both greater exercise intensity and volume may contribute to greater reduction in CapBG.

 Another important finding from the present study was that the timing of exercise can influence the exercise-induced CapBG reduction. Multiple regression analysis revealed that in addition to preexercise CapBG and exercise intervention, medication within 6 hours and food intake within 2 hours of exercise significantly increased the glucose-lowering effect of exercise. ANCOVA with preexercise CapBG as a covariate also confirmed significant effects of food intake and medication after accounting for preexercise CapBG differences (*P* < 0.05). These results suggest that exercise after meal intake can enhance the glucose-lowering effect of exercise and that medication and exercise have an additive effect on CapBG reduction. 

 The finding that food intake accentuates exercise-induced CapBG reduction seen in our study was similar to that of Poirier et al., who reported little changes in plasma glucose when individuals with T2D performed moderate intensity aerobic exercise under fasting conditions while reported significant decreases in plasma glucose under fed conditions [13]. In addition to these findings, however, we propose that the effect of prior meal intake persists during HI-IE. When meals are consumed before exercise, meal-induced hyperglycemia and hyperinsulinemia blunt hepatic glucose output [23], which may have led to a greater imbalance between glucose production and utilization and thereby accentuated the reduction in CapBG. It is also possible that exercise was performed during the period when postprandial glucose was declining, producing synergistic effect with exercise. It can be postulated that exercise performed >6 hours after a meal, on the other hand, lacked the suppressive effect of meal-induced hyperglycemia and hyperinsulinemia on endogenous glucose production and resulted in smaller changes in CapBG. Given the possible pathogenic role postprandial hyperglycemia plays on the risk of diabetic complications [24, 25], performing exercise within 2 hours of meal intake may be a good strategy to attenuate meal-induced hyperglycemia. 

 A magnified CapBG reduction after administration of anti-hyperglycemic medications may not be surprising as they often increase insulin secretion and/or insulin sensitivity in individuals with T2D. Nonetheless, an additive effect of exercise and medication has only been shown with sulfonylurea [26–28] but not consistently with metformin [14, 29], which was taken by the majority of our participants. Studies investigating the effect of exercise plus metformin are limited, and the effect is somewhat equivocal. Since some of our participants were taking a combination of different types of medication, it is difficult to compare our results to existing studies separately investigating the combined effects of exercise and metformin [14, 29] or exercise and sulfonylurea [26–28]. However, when we repeated the same multiple regression analysis after separating the group, according to the medication types, that is, metformin only versus other types such as sulfonylurea and sitagliptin, the effect of medication was no longer significant in metformin group (*P* = 0.085), whereas it remained significant in the combined group (*P* = 0.009). Therefore, although lack of the significant effect of metformin may be simply due to low power, it is also possible that in the present study the effect of medication was mostly attributable to sulfonylurea and sitagliptin, or these medications combined with metformin. No severe hypoglycemia (CapBG < 3.0 mmol/L) was observed in our exercise groups and this may indicate that medication combined with exercise poses a relatively small risk of hypoglycemia in individuals with relatively well controlled T2D (mean A1c = 6.7%), and the risk is likely to be smaller when only metformin is administered.

 An important limitation of the study is that we only evaluated CapBG responses using a single-point measure, and we were unable to determine how blood glucose responded during exercise as well as hours after exercise. Given that improved insulin sensitivity may last 24 hours after a bout of exercise without acute reduction of blood glucose [10], it is possible that exercise bouts resulting in small changes in CapBG observed in the present study led to improved glucoregulation hours following the bout. Nonetheless, because there were no changes in preexercise CapBG over the 12 weeks, the effects of exercise performed on the preceding days on CapBG are considered relatively small. The use of a device such as a continuous glucose monitor may provide a more complete view of glycemic responses.

## 5. Conclusion

 In conclusion, changes in glucose following exercise bouts can be highly variable. Our results showed that preexercise CapBG is the strongest predictor of changes in CapBG induced by exercise accounting for more than 40% of the variability. Furthermore, the glucose-lowering effects of exercise can be accentuated by increasing exercise intensity *per se* without altering exercise volume and/or by increasing exercise duration (volume), as well as by exercising after consuming meals and/or glucose-lowering medications. These results suggest that individuals with less well controlled blood glucose or those who manifest elevated glycemia prior to exercise may benefit more from participating in exercise at least in terms of acute glucose regulation. Lower blood glucose can be achieved with moderate intensity exercise, but including brief bouts of intense exercise and/or prolonging exercise duration can result in a greater glucose reduction if the individual is capable of doing so. These effects of exercise on glycemia can further be enhanced by performing exercise within 2 hours of meal intake and by combining sulfonylurea and sitagliptin. Adding metformin to exercise did not result in significant greater reduction in glucose concentration. However, this result needs to be interpreted with caution given the small sample size used in the present study. The aforementioned variables could be considered by people with T2D and their health care providers to plan exercise intervention and exercise timing to favor greater glycemic improvements. It remains to be seen if the strategy to produce greater acute blood glucose reduction can produce greater longer term improvements in glycemic control as reflected by A1c.

## Figures and Tables

**Table 1:** Baseline characteristics.

	MI-CE	HI-IE	Total	*P* value
*n* (M/F)	4/4	4/3	8/7	
T2D duration (yr)	8 ± 4	6 ± 4	7 ± 5	0.41
Body weight (Kg)	93.9 ± 18.3	80.5 ± 9.9	87.7 ± 16.0	0.10
BMI (Kg/m^2^)	33.1 ± 4.5	28.4 ± 4.1	30.9 ± 4.8	0.06
Hypoglycemic medication				
Metformin alone, *n*	4	4	8	
Metformin and sitagliptin, *n*	1	1	2	
Sulfonylurea and metformin, *n*	2	0	2	
Sulfonylurea and sitagliptin, *n*	1	0	1	
Fasting blood glucose (mmol/L)	7.3 ± 1.7	6.8 ± 0.8	7.1 ± 1.3	0.48
A1c (%)	6.7 ± 0.9	6.6 ± 0.6	6.7 ± 0.7	0.76
VO_2peak_ (mL/kg/min)	18.1 ± 2.7	22.8 ± 5.4^a^	20.1 ± 4.5	0.10

MI-CE: moderate intensity continuous exercise; HI-IE: high intensity interval exercise; VO_2peak_: peak oxygen consumption; A1c: glycated hemoglobin.

*P* value refers to comparisons between HI-IE and MI-CE by independent *t*-test.

Values are presented as mean ± standard deviation. There was no significant difference between HI-IE and MI-CE.

^a^ *n* = 6, VO_2peak_  was not available for one participant.
